# Tailored Adhesion Properties of Acrylate Adhesives on Al Alloys by the Addition of Mn-Al–LDH

**DOI:** 10.3390/polym13091525

**Published:** 2021-05-10

**Authors:** Natasa Z. Tomić, Mohamed Nasr Saleh, Marija M. Vuksanović, Adela Egelja, Vera Obradović, Aleksandar Marinković, Radmila Jančić Heinemann

**Affiliations:** 1 Innovation Center of Faculty of Technology and Metallurgy, Karnegijeva 4, 11000 Belgrade, Serbia; vobradovic@tmf.bg.ac.rs; 2 Structural Integrity & Composites Group, Faculty of Aerospace Engineering, Delft University of Technology, 2628 CD Delft, The Netherlands; 3 “VINČA” Institute of Nuclear Sciences—National Institute of the Republic of Serbia, Department of Chemical Dynamics and Permanent Education, University of Belgrade, Mike Petrovića Alasa 12–14, 11351 Belgrade, Serbia; marija.vuksanovic@vinca.rs (M.M.V.); adela@vin.bg.ac.rs (A.E.); 4 Faculty of Technology and Metallurgy, University of Belgrade, Karnegijeva 4, 11000 Belgrade, Serbia; marinko@tmf.bg.ac.rs (A.M.); radica@tmf.bg.ac.rs (R.J.H.)

**Keywords:** layer double hydroxide, composite adhesive, adhesion, interface, aluminum

## Abstract

The goal of this study was to investigate the effect of the structure of Mn-Al layered double hydroxide (LDH) on the adhesion behavior of composite adhesives on two Al alloys (L3005 and L8079). The composite adhesives were made out of the UV-curing Bisphenol A glycidylmethacrylate/triethylene glycol dimethacrylate (BT) as polymer matrix and the addition of 1, 3, and 5 wt. % of Mn-Al LDH as adhesion enhancers. Adhesion was evaluated by using the micro Vickers hardness testing procedure. The wetting angle of composite adhesives to the Al substrates was measured and compared to the adhesion parameter *b* obtained from the microhardness tests. The highest increase in adhesion was observed for BT with 5 wt. % of Mn-Al LDH on L3005 substrate, which was more than 15 times higher than the adhesion for the neat BT. The morphological segregation of composite adhesives after the contact with Al substrates was examined by optical microscopy and a higher compatibility of Mn-Al LDH particles with L3005 substrate was found. The methods used for the adhesion properties assessment suggested that the Mn-Al LDH was the best adhesion enhancer of the BT matrix for L3005 substrate containing a higher content of Mn and surface hydroxyl groups.

## 1. Introduction

Resin-based composites are commonly used in dental restorative materials due to their light weight, esthetic quality, and biocompatibility [1,2]. Most of the dental composite resins are based on Bis-GMA (bisphenol A-glycidylmethacrylate) [3]. Besides Bis-GMA, other organic monomers which are applied to resin-based dental composites include UDMA (urethane dimethacrylate), TEGDMA (triethylene glycol dimethacrylate), and Bis-GMA (ethoxylatedbisphenol A dimethacrylate). Bis-GMA has a high viscosity, and is frequently combined with low viscosity resin monomers like TEGDMA and EGDMA (ethylene glycol dimethylacrylate) [4]. The mixtures of dimethacrylates are considerably applied to the preparation of composite resins, where TEGDMA is generally used as the diluent to alter the viscosity of the Bis-GMAor UDMA and make their treatment easier [5]. By incorporating TEGDMA and reducing the resin viscosity, a higher filler loading is enabled [6]. The Bis-GMA/TEGDMA composites are characterized by their mechanical properties, which are similar to those of the tooth tissue. These composites are found to have good biocompatibility [4,7]. The polymerization in this system can be initiated by blue light or chemically [8,9]. Light-induced polymerization is important in dentistry as it provides short exposure times and fast curing of the polymer (less than a minute), to ensure that the produced polymer is safe for the patient [10,11].

Traditional composite resins and polyacrylates which contain heavily filled inorganic particle fillers consist of only one continuous phase, the polymer matrix phase [5]. Nanoparticles are generally used as nano reinforcements for the polymer matrix, and they improve the mechanical properties of polymer composites such as hardness, toughness, and modulus of elasticity. Their content can also modify the wear resistance of the composite surface; it can even alter the adhesion of the composite to any surface and the contact angle as well [12,13,14,15]. The filler phase is integrated into the organic matrix of dental composites to tailor various properties to better mimic the properties of the dental tissue that they substitute [16,17]. The stress transfer at the filler–matrix interface, which is influenced by the interfacial properties and surface area of the filler phase, determines the modulus of dental composites [18]. Reduction in size of spherical silica fillers at the same volume content results in better stress distribution and reduction of stress at the filler–matrix interface, leading to improved mechanical properties [18].

Layered double hydroxide (LDH) fillers present a large family of lamellar hydroxides, known as anionic clays [19]. LDHs are a group of inorganic lamellar compounds in which metal hydroxide layers lie on top of each other, and anions and water molecules are located in the interlayer space [20,21]. The general chemical composition of LDH is [M^2+^_1−x_M^3+^_x_(OH)_2_]^−^ (A^n−^)_x/n_·mH_2_O, where M^2+^ and M^3+^ are divalent and trivalent cations, x is the molar ratio of the trivalent cation, and An^−^ is a charge balancing inter-layer anion [22,23]. The M^2+^ and M^3+^ cations coordinated by OH^−^ units occupy the centers of octahedra. These octahedra are connected by edge-sharing to form an infinite sheet of double hydroxide layer [24,25]. The cationic charge created by partial substitution of M^2+^ by M^3+^ cations in the layers is compensated by the presence of hydrated anions between the stacked sheets [26]. The LDH system consists of sheets bonded by covalent forces, where the polymer matrix segments fill up the interlayer gap [27].

Adhesive bonding is an important joining technology in the packaging industry, biomedical applications, dentistry, and microelectronics. Neverthless, polymer composites applied in the industry are characterized by their poor adhesion properties since they have low surface energy and they suffer from the absence of polar functional groups on their surface [28]. Dental composite resins are often modified to improve their adhesive properties [29]. On the contrary, the packaging industry lags behind, especially when it comes to the adhesion properties of the foils’ coatings. Possible ways to characterize the adhesion of thin films and adhesives include: The microhardness test, the scratch test, the impact test, the bending test [30], and the cavitation test [31]. The Vickers hardness technique is commonly used for the examination of composite resins and dentures [32]. Models, through which the assessment of material adhesion via Vickers microhardness can be examined, have been developed [33]. In the characterization of UV-curing adhesives, such as Bis-GMA/TEGDMA-based ones, there is a lack of standard test methods in testing of adhesion properties. A standard test method such as the Bell Peel Test (BPT) considers using two metal adherents, rigid and flexible. Such a joint prevents the UV-curing process of the joint which hinders the use of such a characterization technique. A recent study of bio-based epoxy adhesives, though, managed to validate the potential use of the adhesion parameter *b* by comparing it against results from the BPT [34].

Thus, the objective of this study was to use Mn-Al LDH particles as adhesion enhancers of UV-curing acrylate Bis-GMA/TEGDMA-based adhesives on two aluminum (Al) substrates with different chemical compositions in order to represent an example of the combination of UV-curing acrylate-modified adhesive as a coating for typical packaging foil material. In addition, the compatibility effect of the inorganic adhesion enhancer (Mn-Al LDH) and Al substrate was investigated. Adhesive properties were evaluated by the adhesion parameter *b*, wetting angle, and the Al surface characterization after the removal of the composite adhesive, which was applied as a coating.

## 2. Materials and Methods

### 2.1. Materials

The chemicals MnCl_2_·4H_2_O (Merck-Alkaloid, Skoplje, North Macedonia) and Al_2_(OH)_5_Cl·2.5·H_2_O (Locron L; Clariant company, Muttenz, Switzerland) were used to synthesize the Mn-Al LDH. An appropriate volume of 1.0 mol/L NaOH solutions was used to adjust the pH of the solution. The composite matrix components, Bisphenol A glycidylmethacrylate (Bis-GMA), triethylene glycol dimethacrylate (TEGDMA), camphorquinone (CQ), and ethyl-4-dimethylaminobenzoate (4EDMAB), were all supplied by Sigma—Aldrich. The chemical composition of Al substrates L8079 and L3005 are given in Table 1 and Table 2, respectively. The substrates represent packaging Al foils (100 µm thick) and thus were used as received, without any surface treatment. 

### 2.2. Synthesis of Mn-Al LDH

The Mn-Al layered double hydroxide (Mn-Al LDH) was synthesized by a co-precipitation method, wherein 2.75 g of MnCl_2_ 4H_2_O and 1.25 g of Al_2_(OH)_5_Cl 2,5·H_2_O (molar ratio of 3:1) was dissolved one after the other in 100 mL deionized water with continuous stirring. The pH of the resulting turbid solution was increased to 10 by adding 1 mol/L NaOH. The resulting slurry was aged for 24 h, centrifuged at 6000 r/min for 10 min, and washed with de-ionized water repeatedly until the solution pH was neutral. The wet solid was dried at 80 °C for 24 h to obtain Mn-Al-LDH.

### 2.3. Preparation of Composite Adhesives

Bis-GMA 49.5%, TEGDMA 49.5%, CQ 0.2%, and 4EDMAB 0.8%, were mixed and polymerized under UV light for 3 min in order to obtain the acrylic polymer matrix (BT). Composite adhesives were made with 1 wt. %, 3 wt. % and 5 wt. % content of Mn-Al LDH filler. The adhesives were applied by the drop coating method on Al foils [35]. The method considers the application of a thin cap after applying one drop of monomer solution to an Al surface. The final thickness of the applied adhesive was controlled by the weight of the glass cover placed over the drop.

A decision on the share of particles for the preparation of composite adhesives was made based on the previous experience [29], because already at 5 wt. % of particles the agglomerates appear. Higher percentages are causing also a difficult sample preparation. Thus, the optimum dispersion in BT composites was only found in the range of 1–5 wt. % of fillers addition. Besides, the literature about LDH in dental composites used only 3 and 5 wt. % of LDH fillers [36].

### 2.4. Material Characterization

#### 2.4.1. Structural Characterization

Structural characterization of LDH particles was performed by analyzing the crystal phases of Mn-Al LDH by an Ital Structure APD2000 X-ray diffractometer in a Bragg–Brentano geometry using CuKα radiation (λ = 1.5418 Å) and step-scan mode (range: 20°−80° 2θ, step-time: 0.50 s, step-width: 0.02°). Besides, the Fourier-transform infrared spectroscopy (FTIR) was used for the characterization of the chemical composition of the interface surfaces of both the Al substrate and polymer adhesives with and without Mn-Al LDH fillers. Tests were performed using a Nicolet 6700 spectrometer (Thermo Scientific, Waltham, MA, USA) in the attenuated total reflectance (ATR) mode with a single bounce 45 °F Golden Gate ATR accessory with a diamond crystal, and an electronically cooled DTGS detector. FTIR spectra were obtained at 4 cm^−1^ resolution with ATR correction. The Nicolet 6700 FTIR spectrometer was equipped with OMNIC software and the spectra were recorded in the wavelength range from 2.5 μm to 20 μm (i.e., 4000–500 cm^−1^) [37].

#### 2.4.2. Characterization of Morphology

The morphologies of the Mn-Al LDH fillers and adhesives were examined using a field emission scanning electron microscope (FE-SEM), MIRA3 TESCAN, Brno–Kohoutovice, Czech Republic, operated at 3 kV. SEM images of the Mn-Al LDH fillers were used to determine the size distribution of particles. The tools of the software package Image-Pro Plus 4.0 (Media Cybernetics, Rockville, MD, USA) were used. Elemental analysis on interfaces was done by using electronic dispersive spectroscopy (EDS) by JEOL 5800 JSM (JEOL, Akishima, Japan).

#### 2.4.3. Adhesion Assessment

The adhesion strength was estimated by the adhesion parameter *b*, which is obtained by the microhardness test method [38]. The parameter *b* represents the ratio of the radius of the indented plastic region to the indented depth, *b* = *r*/*h*, which varies depending on the combination of the film (in our case it is an adhesive) and the substrate. This technique was recently proved to be useful as a fast and reliable testing method in the adhesive selection process when compared to standard BPT [34]. The essence of this technique lies in the differences captured in the indent geometry. A strong adhesion at the interface results in distortion of the plastic zone, while a weak adhesion is characterized by strain discontinuity across the interface.

The method was done using micro Vickers hardness (HV) tester Leitz, Kleinharteprufer DURIMETI. The equation used to calculate the Vickers number is:

(1) $$VHN=2\cos\frac{22{}^{\circ}P}{d^{2}}=\frac{1.8544P}{d^{2}}$$

where *P* (kgf) is the applied load, and *d* (mm) is the length of the indentation diagonal [33].

The hardness of the samples was measured with different loads (50, 100, 200, 300, and 500 gf) with a 25 s duration, whereby for each loading three indents were performed. Images of indentation marks were captured by the optical microscope (Carl Zeiss-Jena, Jena, Germany, NU2) and were used to obtain the diagonal lengths using the Image-Pro Plus program.

In the model [39], the mean length of the diagonals *D* is used as an input parameter to match the composite hardness relation by the model indicated by the equation on the depth indentation:

(2) $$H_{c}=A+B\frac{1}{D}+C\frac{1}{D^{m+1}}$$

where the fitting parameters are *A*, *B*, *C*, the composite hardness is *H_c_* and the parameter m (power index) has values in the range of 1–2 (*m* = 1.8 is for the case of a soft adhesive on a hard surface) [40]. The equation used to measure the hardness of the adhesive is given by:

(3) $$\;H_{f}=A\pm \sqrt{\frac{{\left[m\left|B\right|/\left(m+1\right)\right]}^{m+1}}{m\left|C\right|}}$$

Equation (3) is used for the measurement of the critical reduction in depth *b* [41]:

(4) $$\Delta H=\left[\frac{7\cdot \left(m+1\right)\cdot \left(H_{s}-H_{f}\right)}{m\cdot b}\right]\cdot \frac{t}{d}$$

The indentation depth is expected to be D/7, and the adhesive thickness is *t*. Values: *H_s_*, *H_c_*, and *d* are measured by direct experimental measurement. *H_f_* is calculated as a function of *h*/*t* by fitting the experimental results for *H_c_*. Finally, the value of parameter *b*, considered as adhesion indicator, is determined by the slope of the linear fit of *dH* = *f* (*t*/*d*) from Equation (4).

The evaluation of the adhesion of the BT and composite adhesives on the aluminum foil was followed by determining the wetting angle. A drop of the composite mixture was placed on an aluminum substrate and polymerized using a UV lamp. Optical microscope (Delta Smart 5MP Pro digital material inspection microscope) images of the samples were taken and the angles of contact of the adhesives with the metal surface were measured using image analysis software.

## 3. Results

### 3.1. The Microstructure of Mn-Al LDH Fillers

**Figure 1:**
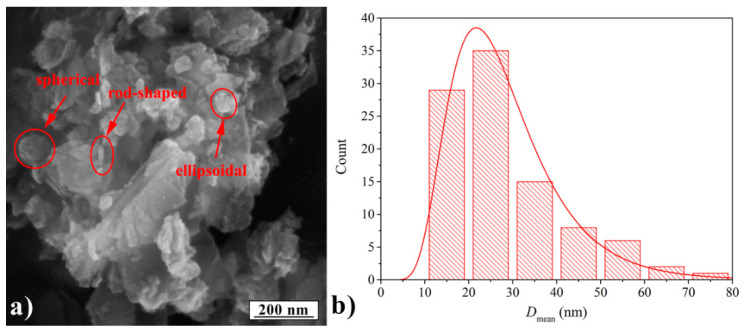
(**a**) FE-SEM micrograph of Mn-Al LDH filler and (**b**) lognormal diameter distribution.

The morphology of Mn-Al LDH samples was examined using FE-SEM, Figure 1a. The diameter distribution analysis was carried out with image analysis tools, and the obtained results are shown in Figure 1b and summarized in Table 3.

Based on the Mn-Al LDH diameter distribution, it can be seen that most particles have a diameter between 20 and 30 nm. The FE-SEM images show that the Mn-Al LDH samples have ellipsoidal, spherical, and rod-shaped particles. The particle size was found to be in the range of 10–50 nm, and the morphology was similar to the morphology of Mn_3_O_4_ following XRD patterns [42]. It can be also seen that the particles were bound to each other forming an agglomerate, suggesting strong hydrogen bonding, which can be very beneficial for establishing good adhesion.

### 3.2. The XRD Analysis of Mn-Al LDH Crystal Structure

**Figure 2:**
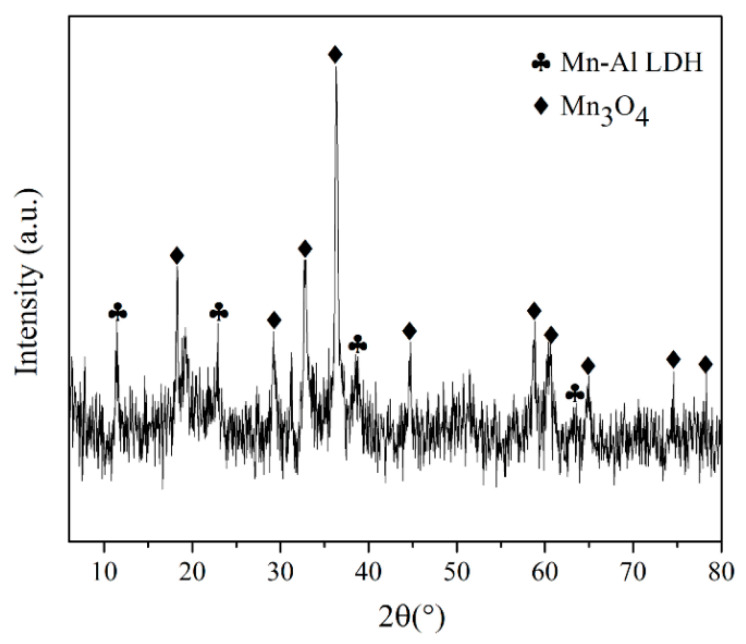
XRD patterns of Mn-Al LDH fillers.

Figure 2 shows the XRD patterns of Mn-Al LDH in the range of 5°–80°. XRD analysis indicated that two main compounds—Mn-Al LDH and Mn_3_O_4_. The diffraction peaks at 11.3°, 22.8°, 38.6°, and 63.4° correspond to the planes (003), (006), (015), (113) of a typical layered structure. The XRD results show that MnCl_2_ and Al_2_(OH)_5_Cl were successfully transformed into the LDH phase [43]. The d_003_ value of Mn-Al LDH was 7.76 Å. The cell parameter “c” calculated from c = 3·d_003_ is 2.328 nm.

### 3.3. Adhesion Assessment

**Figure 3:**
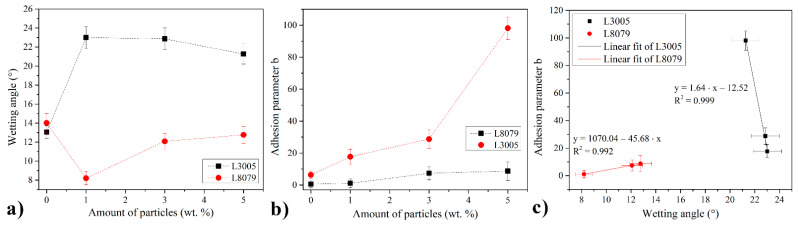
(**a**) Wetting angles of BT with different amount of Mn-Al LDH for the two substrates L3005 and L8079, (**b**) the adhesion parameter *b*, and (**c**) linear fit of adhesion parameter *b* vs. wetting angle scatter diagram for the two substrates L3005 and L8079 (Note: In Figure 3c the data for neat BT were omitted due to the different adhesion mechanism with Al substrates as compared to composite adhesives.).

The wetting angle measurements of adhesive droplets on L3005 and L8079 substrates are presented in Figure 3a. They show the opposite behavior over the two substrates. The wetting of BT adhesive was similar for both substrates (13.04° for L3005 and 14.02° for L8079), but the addition of Mn-Al LDH changed the trends. The presence of 1 wt. % of particles decreased the wetting on L8079 suggesting the favorable interactions between the substrate and the composite adhesive. Further addition showed the increase of the wetting angle indicating possible adverse effects caused by the addition of Mn-Al LDH in BT for L8079. On the contrary, the addition of 1 wt. % of Mn-Al LDH caused the increase of the wetting angle, which can suggest a higher amount of interaction within the composite adhesive itself in the contact with L3005. The value of the wetting angle was still low, which is desirable for good adhesion. Further increase of Mn-Al LDH amount lowered the value of the wetting angle indicating favorable interactions with L3005 substrate. The wetting angle is a sum of effects of different parameters, and its low values are a good precondition to establishing strong adhesion but which is not necessarily the case. Thus, the adhesion properties were further assessed by the adhesion parameter *b*.

A Chen-Gao mathematical model was used to quantify the factor *b*, which describes the degree of adhesion between the substrate and the film, through the use of microhardness measurements [42]. In Figure 3b, the dependency of the adhesion parameter *b* on the amount of Mn-Al LDH in the composite adhesives is shown. It was observed that the composition of Al alloy had a significant effect on the adhesion of composite adhesives. L3005 substrate with a higher amount of alloying elements showed higher improvement of adhesion than L8079. Nevertheless, the introduction of Mn-Al LDH particles into BT showed a positive effect on the improvement of the adhesion to both substrates. The highest increase in adhesion was observed for BT+5 wt. % of Mn-Al LDH on L3005 substrate, which was more than 15 times higher than the adhesion for the neat BT. Such an enormous increase in adhesion can be prescribed to better compatibility between the L3005 and the Mn-Al LDH in the composite adhesive than for the L8079. The reason for that was that the main alloying element in L3005 is Mn, which forms a stable form of manganese oxide in the contact with air, called hausmannite (Mn_3_O_4_). Mn_3_O_4_ exhibits strong adsorption properties [44] and as such establishes strong interactions with organic and inorganic molecules and ions. So, the presence of Mn_3_O_4_ in LDH only was not enough for good adhesion but the matched compatibility with the substrate.

Figure 3c shows the linear fit of adhesion parameter *b* vs. wetting angle scatter diagram for the two substrates L3005 and L8079. Very good and reliable data fitting indicates a significant and positive impact of Al-Mn LDH fillers for L3005 substrates and negligible effect for L8079 substrate. Besides, when the statistical error was considered for the L8079 substrate, there is almost no further improvement of the adhesion with the addition of more particles. Only the wetting angle increased which indicated the adverse effect on the wetting and potential defect formation at the interface. 

### 3.4. Microstructural Analysis

**Figure 4:**
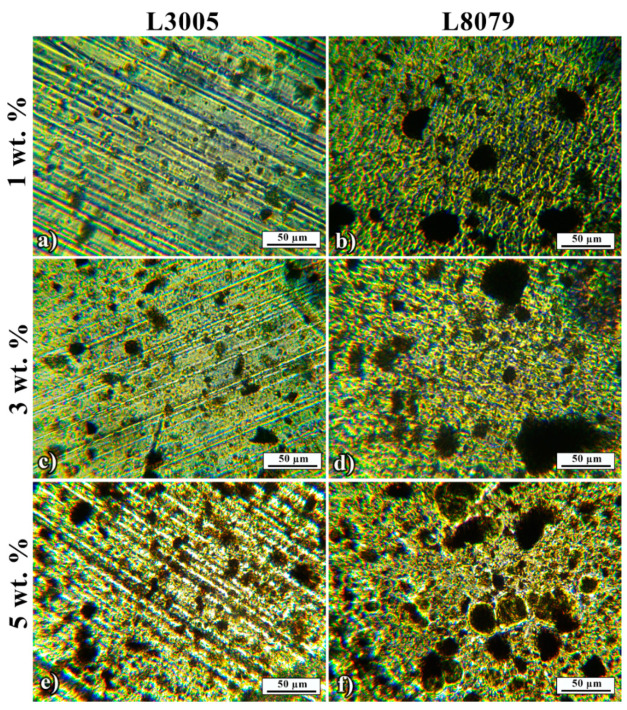
Optical micrographs of composite adhesives: (**a**) 1 wt.% Mn-Al LDH removed from L3005 and (**b**) from L8079 surfaces; (**c**) 3 wt.% Mn-Al LDH removed from L3005, and (**d**) from L8079 surfaces; (**e**) 5 wt.% Mn-Al LDH removed from L3005, and (**f**) from L8079 surfaces.

**Figure 5:**
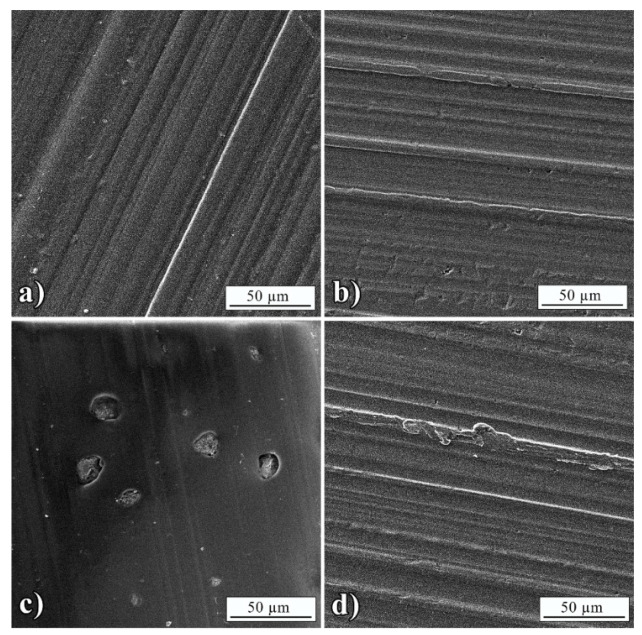
FE-SEM micrographs of: (**a**) Peeled BT interface with L3005, (**b**) L3005 interface after removing BT, (**c**) peeled BT+5 wt.% Mn-Al LDH interface, and (**d**) L3005 interface after removing BT+5 wt.% Mn-Al LDH.

An interesting phenomenon can be observed on optical micrographs of composites adhesives BT+ Mn-Al LDH removed from the two substrates L3005 and L8079, in Figure 4a–f. The same composite adhesive applied to different surfaces exhibited different intermolecular interactions within the adhesive itself. More precisely, the arrangement of Mn-Al LDH fillers in a polymer matrix was affected by the interactions with the substrate. Figure 4a shows composite adhesive with the addition of 1 wt. % of particles to BT removed from L3005 having a uniform distribution of the smallest aggregates when compared to higher percentages. The application of the same composite to the L8079 substrate caused the formation of large aggregates due to the lower compatibility with the substrate. The size of the aggregates for L3005 substrate with 3 wt. % (Figure 4c) and 5 wt. % (Figure 4e) of Mn-Al LDH is slightly increasing due to the increased amount of particles leading to a higher extent of interactions. Aggregates from L8079 substrates in all composites, i.e., BT + 1 wt. % (Figure 4b), 3 wt. % (Figure 4d) and 5 wt. % (Figure 4f), are significantly larger than the ones from the L3005 substrate. Besides, there is no indication that any of the composites showed good compatibility with L3005. Thus, the compatibility of L3005 and Mn-Al LDH contributed to the formation of smaller aggregates than in the case of L8079. 

Table 4 presents the size distribution of the Mn-Al LDH aggregates observed in Figure 4. The size of aggregates in the case of L3005 with 5 wt. % of Mn-Al LDH was 60% lower according to a *D*_mean_. The lower standard deviation and range of the aggregate size (*D*_max_ − *D*_min_) confirmed the improved L3005/Mn-Al LDH system compatibility.

**Figure 6:**
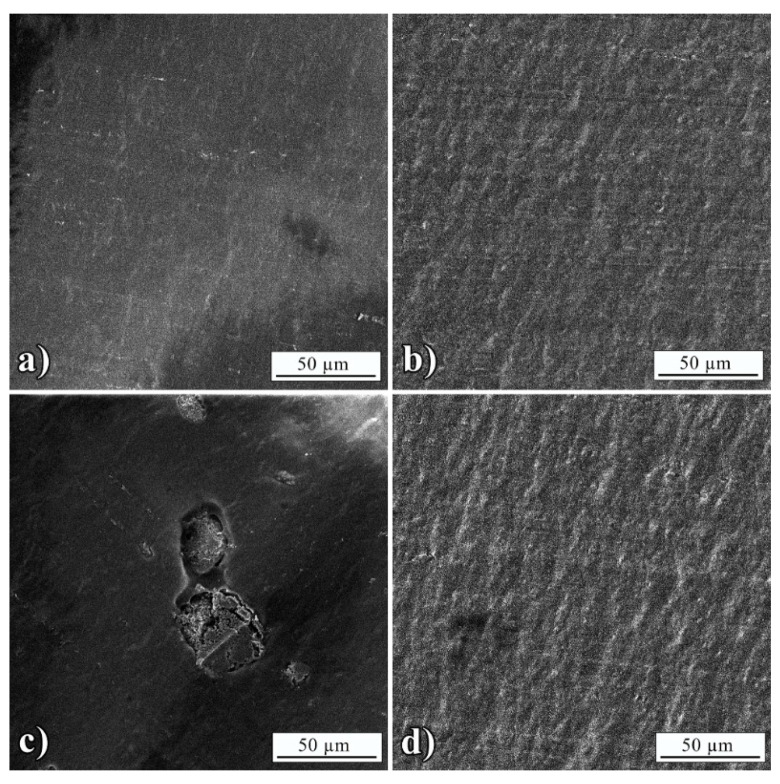
FE-SEM micrographs of: (**a**) Peeled BT interface with L8079, (**b**) L8079 interface after removing BT, (**c**) peeled BT+5 wt.% Mn-Al LDH interface, and (**d**) L8079 interface after removing BT+5 wt.% Mn-Al LDH.

Detailed analysis of debonded surfaces was studied by FE-SEM, which is presented in Figure 5 and Figure 6. Morphology of BT interface (Figure 5a) matched the L3005 interface (Figure 5b) showing complete adhesive failure with no remained adhesive on Al substrate. The adhesion of BT was slightly improved with the addition of Mn-Al LDH, which was observed by traces of remained composite adhesive indicating partial cohesive failure (Figure 5d). Figure 5c showed Mn-Al LDH aggregates in the size range previously measured from optical micrographs (Table 4).

The morphology of the BT interface (Figure 6a) again matched the L7089 interface (Figure 6b) with no observed remained adhesive. The improvement of BT adhesion with the addition of Mn-Al LDH could not be noticed since the morphology of L8079 remained the same as in the case of neat BT (Figure 6d). Figure 6c showed Mn-Al LDH aggregates in the same size range measured from optical micrographs (Table 4).

### 3.5. FTIR Characterization of Al Alloys, Composite Adhesives, and Interfaces 

**Figure 7:**
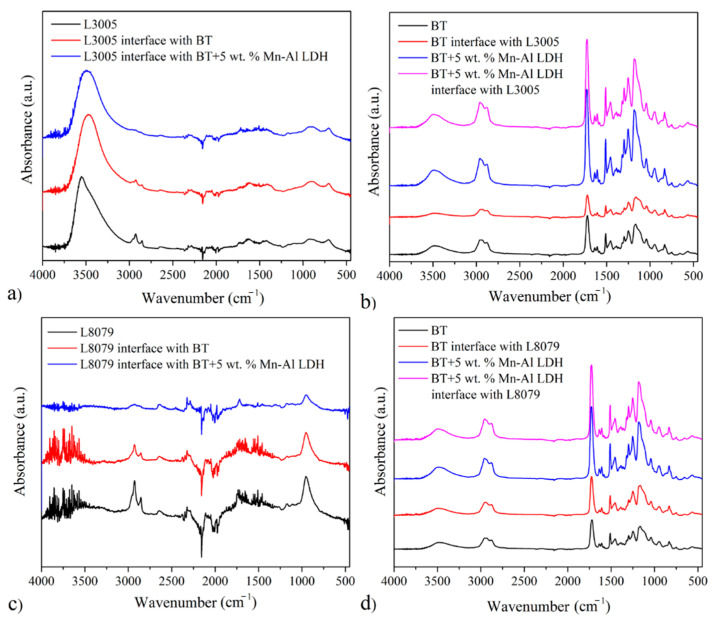
FTIR analysis of (**a**) L3005 and its interfaces, (**b**) BT and BT composite adhesives and their interfaces with L3005, (**c**) L8079 and its interfaces, and (**d**) BT and BT composite adhesives and their interfaces with L8079.

FTIR analysis of composite adhesives with and without Mn-Al LDH, aluminum alloys, and their interfaces was performed to investigate the presence of composition and chemical species on materials surfaces, Figure 7. Comparison of FTIR spectra of neat substrates showed that L3005 possessed a significant amount of available surface hydroxyl groups, observed at 3500 cm^−1^, Figure 7a. As opposed to this, L8079 did not show the presence of any hydroxyl group, Figure 7c. Both substrates showed the presence of C-H vibrations noticed around 2850 cm^−1^ suggesting the presence of oily impurities. Their content decreased after BT and BT+5 wt. % Mn-Al LDH were applied since they can be absorbed by LDH and react in cross-linking reactions of BT matrix. BT, its composite, and their interfaces on L3005 and L8079 are shown in Figure 7b,7d, respectively. 

At ~3490 cm^−1^, the characteristic peaks for O–H stretching vibration were observed. The peaks observed in the 2956–2875 cm^−1^ range were due to the Bis-GMA/TEGDMA matrix aliphatic C–H stretch [29]. The peak at 1734 cm^−1^ can be assigned to the polymer matrix’s carbonyl C=O group vibration. The peak at 1638 cm^−1^ (assigned to the aliphatic unsaturated chains) and the peak at 1609 cm^−1^ (assigned to the aromatic structure) [45] were confirmed by the unsaturated C=C double bonds in the composites. Methyl (isopropylidene moiety in Bisphenol A) and methylene group deformation vibrations are observed at 1511 and 1453 cm^−1^, respectively. Valence C–O bond vibration modes were shown as alternating bands at 1252, 1181, and 1038 cm^−1^ in ester, ether, and phenolic pairs, both asymmetric and symmetric. The absorption peaks below 1000 cm^−1^ were due to unsaturated C–H bonds from out-of-plane bending vibration modes [45]. The spectra showed that with the addition of Mn-Al LDH to BT, the amount of hydroxyl group’s content increased, which was observed by the increased intensity of the peak found at 3500 cm^−1^. Other peaks characteristic for BT remained the same for both of the substrates. The higher amount of the surface hydroxyl groups are favorable for establishing hydrogen bonding interaction with an adhesive containing Mn-Al LDH. The lower amount noticed for L8079 can cause an adverse effect with the addition of LDH particles.

### 3.6. EDS Analysis 

Detailed analysis of the substrate interfaces was performed to determine the presence of cohesive failure by EDS elemental analysis, Table 5. The L3005 interface with BT with a magnification of 200× showed an amount of Mn similar to the producer’s specification (Table 2). The content of O was observed due to the surface hydroxyl groups or the formed oxides, which was almost two times higher for the L3005 interface with BT than for L8079. In the case of L3005 interface with BT+5 wt. % Mn-Al LDH with a magnification of 200×, no significant changes were observed. With an increased magnification of 50 k× on the observed remains, the elemental composition significantly changed. The appearance of C indicated the presence of BT on the surface, while the high amount of Mn (10.5%) showed their affinity with the L3005. L8079 interface with BT with a magnification of 200× showed the same amount of Fe as per the producer’s specification (Table 1). The L8079 interface with BT+5 wt. % Mn-Al LDH with a magnification of 200× did not show any notable changes when compared to the neat BT. With an increased magnification of 50 k× on the observed remains, L8079 showed a higher amount of C and a much lower amount of Mn. This observation coincides well with all the previous results suggesting better compatibility of Mn-Al LDH particles with L3005 substrate.

## 4. Conclusions

The Mn-Al layered double hydroxide (Mn-Al LDH) was synthesized by a co-precipitation method with a particle size in the range of 10–50 nm and morphology similar to the morphology of Mn_3_O_4_. They were used as adhesion enhancers of adhesives based on acrylate BT matrix on two different Al alloys, L3005 and L8079. Two techniques were used to assess the adhesion between the composite adhesives and the metal surfaces: Microhardness combined with theoretical simulations and measurements of the wetting angle. With an increase in particle content, the adhesion improved. The morphological segregation of Mn-Al LDH particles in composite adhesives after the contact with Al substrates was examined by optical microscopy. Particle distribution and aggregate size indicate higher compatibility of Mn-Al LDH particles with L3005 substrate. The methods used for the adhesion strength assessment suggested that the Mn-Al LDH was the best adhesion enhancer of the BT matrix for L3005 substrate containing a higher content of Mn and surface hydroxyl groups. The results of this study indicate that LDH particles, obtained simply and inexpensively, can be used as adhesion enhancers of acrylate adhesives, films, or coatings. Nevertheless, their power of adhesion enhancement can be tailored by a proper selection of LDH cations to improve their compatibility with the substrate.

## Figures and Tables

**Table 1:** Chemical composition and mechanical properties of Al substrate L8079.

Si (%)	Fe (%)	Mg (%)	Al (%)
0.11	1.0	0.001	98.8

**Table 2:** Chemical composition of Al substrate L3005.

Si (%)	Fe (%)	Cu (%)	Mn (%)	Mg (%)	Cr (%)	Zn (%)	Ti (%)	Pb (%)	Al (%)
0.19	0.6	0.11	1.2	0.55	0.01	0.02	0.03	0.002	97.288

**Table 3:** The grain size distribution of Mn-Al LDH particles.

Mean (nm)	Minimum (nm)	Maximum (nm)	Standard Deviation (nm)	Variance (nm)
28.39	12.51	74.32	13.25	175.53

**Table 4:** The size distribution of Mn-Al LDH aggregates after the contact with Al substrate.

Sample	*D*_max_ (µm)	*D*_min_ (µm)	*D*_mean_ (µm)	Standard Deviation (µm)
L3005/BT+1 wt.% Mn-Al LDH	14.18	1.86	5.34	2.58
L3005/BT+3 wt.% Mn-Al LDH	26.94	2.79	9.71	5.83
L3005/BT+5 wt.% Mn-Al LDH	38.80	4.24	10.41	6.90
L8079/BT+1 wt.% Mn-Al LDH	48.94	4.52	16.87	11.83
L8079/BT+3 wt.% Mn-Al LDH	96.99	5.12	18.08	18.88
L8079/BT+5 wt.% Mn-Al LDH	56.42	5.73	26.13	10.93

**Table 5:** EDS analysis of Al interfaces.

Sample/Elements	C (%)	O (%)	Al (%)	Mn (%)	Fe (%)
L3005 interface with BT, 200×	-	3.17	95.36	1.01	0.45
L3005 interface with BT+5 wt. % Mn-Al LDH, 200×	-	3.28	95.23	1.04	0.46
L3005 interface with BT+5 wt. % Mn-Al LDH, 50 k×	10.4	16.4	61.82	10.5	0.78
L8079 interface with BT, 200×	-	1.83	97.12	-	1.02
L8079 interface with BT+5 wt. % Mn-Al LDH, 200×	-	1.76	97.26	-	0.99
L8079 interface with BT+5 wt. % Mn-Al LDH, 50 k×	14.8	14.6	64.02	5.75	0.82

## Data Availability

Not applicable.
